# Integration of sex and gender in cardiovascular medicine: a scientific and clinical imperative

**DOI:** 10.1007/s12471-023-01817-9

**Published:** 2023-09-08

**Authors:** Maryam Kavousi, Jeanine Roeters van Lennep

**Affiliations:** 1 Department of Epidemiology, Erasmus MC, University Medical Centre Rotterdam, Rotterdam, The Netherlands; 2 Cardiovascular Institute, Department of Internal Medicine, Erasmus MC, University Medical Centre Rotterdam, Rotterdam, The Netherlands

The past two decades have witnessed a growing understanding of sex-based differences in cardiovascular diseases. Age-related and sex-based differences in cardiovascular risk and varying associations of traditional cardiometabolic risk factors with cardiovascular diseases have been active areas of research. While associations of several key traditional cardiometabolic risk factors with cardiovascular outcomes are stronger in women, traditional risk factors account for a smaller proportion of the total cardiovascular disease risk in women. Such sex differences in residual risk could be attributed to insufficiency in defining and measuring the risk factors. More importantly, they could also point towards yet unidentified, sex-specific, risk factors [1, 2]. In line with this, recent guidelines for cardiovascular disease prevention highlight several sex-specific conditions including obstetric conditions (i.e. pregnancy and its related complications), non-obstetric conditions including premature menopause and endocrine disorders (e.g. polycystic ovary syndrome), and erectile dysfunction to be associated with future cardiovascular events [3]. In parallel, there is increasing understanding regarding the links between cardiovascular conditions and the genome, epigenome, transcriptome, and metabolome. This calls for studies to investigate sex differences in genetic architecture and heritability, epigenetic modifications, transcriptomic profiles, and metabolomic signatures [4, 5]. Such studies could also contribute to filling the gap in our understanding of sex-specific ranges or thresholds for established and novel cardiovascular biomarkers.

While sex is a biological construct referring to anatomical characteristics and physiological features, gender is a multidimensional construct comprising psychological, social, and cultural identities, behaviours, norms, and roles. Whereas the awareness of gender differences is important for the prevention and management of cardiovascular diseases, gender-related variables remain to be defined for most cardiovascular diseases in the future. Differences in cardiovascular disease manifestation, in particular for ischaemic heart disease, stem from a complex interplay between sex-based pathophysiology and gender-based behaviours. Differences in the management of cardiovascular diseases are also likely due to a combination of sex-based biological differences in response to cardiovascular interventions as well as gender-based differences in patterns of prescribing or titrating therapies and in adherence. More importantly, some of the available cardiovascular drugs as well as interventions and therapies might be functionally insufficient or incompatible for the treatment of female-specific cardiovascular conditions. More recent advances in cardiovascular medicine and the potential opportunities for artificial intelligence in screening and management of cardiovascular diseases also mandate better understanding of the interaction between sex and gender in cardiovascular conditions. To fulfil their promise to impact public health and clinical medicine, the artificial intelligence and digital tools could be programmed to specifically incorporate sex- and gender-related factors to narrow the disparity gap, and thus improve cardiovascular prediction and prevention, cardiovascular disease management, and patient outcomes [6].

The scientific and clinical imperative for sex- and gender-informed perspectives is increasingly becoming clear. Notwithstanding the achievements to date, we are still at the beginning stages of appreciating sex differences in cardiovascular biology. Sex differences are a cumulative effect of genetics, epigenetics, transcriptomics, proteomics, hormonal influences, and structural and morphological variations, among others. Additional complexity arises from the impact of environmental exposures and hormonal interactions. Gender-related norms and social factors add another, thus far not well-established and investigated, layer of complexity. Sex and gender diversity is another area in urgent need of new knowledge. To help stimulate and drive further advances in the cardiovascular field, implementation of a sex- and gender-specific lens across all of our research and clinical work is necessary.
